# Pre-hospital delay in patients with ischemic stroke in the Fann Teaching Hospital, Dakar, Senegal in 2020

**DOI:** 10.11604/pamj.2022.41.79.30191

**Published:** 2022-01-28

**Authors:** Michel-Arnaud Saphou Damon, Anna Modji Basse, Adjaratou Dieynabou Sow, Prisca-Rolande Bassole, Marième-Soda Diop-sene, Franck-Ladys Banzouzi, Mame Maïmouna Diaw Santos, Kamadore Toure

**Affiliations:** 1 Neurology Clinic Ibrahima Pierre Ndiaye, Fann Teaching Hospital, Dakar, Senegal,; 2 Department of Neurology, El Hadj Ibrahima Niasse Private University-College of Medicine Saint Christopher Ibar Mar Diop, Dakar, Senegal,; 3 Department of Public Health, University of Thiès, Thiès, Senegal

**Keywords:** Pre-hospital delay, acute ischemic stroke, sub-Saharan Africa, neurovascular unit

## Abstract

**Introduction:**

stroke is a cerebrovascular disease. Early reperfusion in neurovascular units can reduce its morbidity and mortality. Even when neurovascular units exist, patients usually arrive late in the emergency department. to the purpose of this study was to determine prehospital delay in patients with acute ischemic stroke and associated factors.

**Methods:**

we conducted a retrospective cross-sectional study in the neurology department of the Fann University Hospital in Dakar from January 1^s^^t^ to June 30^th^, 2020. We included patients younger than 80 years seen in the emergency unit for ischemic stroke. The median time to presentation was calculated based on the time of stroke onset and that of arrival at the hospital. Multivariate analysis was used to determine factors associated with prehospital delay.

**Results:**

a total of 56 patients were enrolled, among whom 58.6% arrived at the hospital in less than 3 hours. Of them, 37.5% presented to a level 3 or 4 hospital first. Less than 34% of our patient presented to a level 2-3 hospital in less than 3 hours. Based on bi- and multivariate analysis, being married (OR = 7.2 [CI à 95%: 1.5 - 35.8]), being a female (OR = 5.5 [CI à 95%: 1.5 - 19.8]) and having stroke during week days (OR = 4.3 [CI à 95%: 1.3-13.9]) were associated with prehospital delay.

**Conclusion:**

most of our patients arrived late at a level 2 or 3 hospital. Being a married woman increased the risk of late arrival. This study highlights the importance of improving awareness in order to increase the proportion of patients potentially eligible for revascularization.

## Introduction

Ischemic stroke is defined by WHO and ICD-11 as a neurovascular condition [1,2]. This definition was revised by the American Heart Association and the American Stroke Association in 2013 as an episode of neurological dysfunction caused by focal cerebral, spinal, or retinal infarction [3,4]. In Dakar, stroke is the leading neurological condition requiring hospitalization. Stroke represents more than 30% of hospitalizations and is responsible for nearly two-thirds of deaths observed in the neurology department [5]. Its incidence is estimated at 1-2% in the general population in Senegal [6,7]. Early identification of the signs of stroke by the patient or the bystanders is critical to timely access to appropriate care´s facilities [8]. Early presentation helps reduce stroke´s morbidity, which can be extremely variable [9], and mortality. Patients who use prehospital emergency medical services are more likely to receive thrombolytic therapy with a reduction in stroke morbidity and mortality [10]. Neurovascular units (NVUs) should therefore be widely implemented to effectively manage stroke [11]. The purpose of our study is therefore to determine the onset-to-door's delay for patients after stroke and to analyze factors associated with this delay.

## Methods

**Study design and setting:** we conducted a cross-sectional study, at the Neurology Clinic Ibrahima Pierre Ndiaye (IPN) of Fann Teaching Hospital, Dakar Senegal over the period from January 1^st^ to June 30^th^, 2020. The Neurology Clinic Ibrahima Pierre Ndiaye includes an outpatient unit, an inpatient unit, a computed tomography (CT) scan and a Neuropsychology Unit. The outpatient unit is the place for routine consultations and neurological emergencies. Neurological emergencies are either referred by the hospital's emergency reception service (ERS) or referred from another public or private health care facility. Consultations are provided by residents in neurology under the supervision of senior neurologists. Patients seen in the neurological emergency department are either hospitalized in the neuro-intensive care unit or in the ward, or are hospitalized in the day-care ward or are treated on an outpatient basis. A specific appointment is scheduled for each patient when he or she returns home.

**Participants:** the study included all patients under 80 years seen in the emergency service of the neurology outpatient department for management of a sudden neurological deficit. Brain imaging (computed tomograph (CT) or magnetic resonance imaging (MRI)) results were consistent with recent ischemic stroke. Included patients or their relatives had signed informed consent. Patients with hemorrhagic strokes, cerebral venous thrombosis, and those who came for a follow-up visit after stroke were not included. All patients with ischemic stroke for whom the date and time of stroke onset or the date and time of presentation to a level 2-3 hospital facility could not be specified by the patient or family members were excluded. Patients who wished to withdraw from the study were also excluded. Patients' itinerary was the one promulgated by the Ministry of Health and Social Action in accordance with the health pyramid. In case of illness, patients should first go to a primary care facility. For conditions that require a more equipped medical facility, patients should resort to a secondary health structure or even a level 3 health structure. Data collection was done using a questionnaire. The questionnaire was developed by the research team. It was administered directly to the included patient or his or her accompanying person by the investigator. The approximate time of onset of the deficit as well as the time of arrival at the emergency room was asked by the investigator to the patient or the patient's companion who witnessed the accident. The time of the imaging (CT/MRI) was reported on the images. Information about sociodemographic characteristics, social network and prehospital transport were also collected directly from the patient, his or her family and friends. A number was assigned to each patient according to the time of admission in the study and according to a specific coding that was known only to the investigator.

**Variables:** the variables studied were defined as follow;

**Timeframes:** the various timeframes expressed in hours have as a starting point the time of occurrence of the deficit.

**Late referral:** referral beyond 3 hours was considered a late referral. We drew inspiration from the work of Bassong *et al*. realized in the service in 2012. This enabled us to better compare our results [12].

**Level 2-3 facility:** any hospital facility with brain imaging tools for the diagnosis of stroke (CT scan or MRI).

**Nearby health center:** we have grouped together under the name of nearby health center all the public health structures belonging to the primary sector of the health pyramid of Senegal.

**Primary care facility:** the first facility or entity to which a patient has gone after the onset of an ischemic stroke.

**Day of onset:** were split into two groups as follows, weekday (all days from monday to friday) and weekend (Saturday and Sunday).

**Statistical analysis:** collected data were entered on a computer with Excel Office 2016 software and then analyzed with STATA SE 15.1 software. Uni, bi and multivariate analyses were performed. Bivariate analysis allowed us to calculate the frequencies for qualitative variables and the central tendency and dispersion parameters for quantitative variables. Then we compared the onset-to-door delay according to the independent variables by the Chi-square test. The difference was considered as statistically significant when p-value was strictly less than 0.05. Multivariate analysis allowed us to identify factors independently associated with onset-to-door delay using the binary logistic regression method. All variables with p values less than or equal to 0.25 were used to model delay in presentation to a level 2-3 health structure. It was applied to top-down modeling. Adjusted odds ratio (AOR) with their 95% confidence interval (CI) were determined for each variable retained in the final model. The goodness of fit for the model was established using the Hosmer and Lemeshow test to verify its adequacy.

**Ethical consideration:** the approval to undertake the study was obtained by the Director of the study from Ethical Review Committee of FANN Teaching Hospital. Written informed consent was obtained from each participant before being recruited into the study.

## Results

**Participants and descriptive data:** we evaluated 178 patients with abrupt onset neurological deficit. After the brain imaging test, 67 subjects with acute ischemic stroke were included. Of these patients, 11 were secondarily excluded because they could not specify the date and time of stroke onset or the date and time of presentation to a level 2-3 structure. Therefore, only 56 patients were included in the study. The mean age of patients was 48.2 ± 13.2. The median was 45 years (ranging between 25 years and 78 years). The majority of our patients were between 40-49 years of age. We observed a male predominance (30 males, 26 females) with a sex ratio of 1.15. More than half of patients or 53.6% had, at least, completed primary education level (33.9%). Married subjects accounted for 66.1% of patients. The vast majority of patients, 83.9%, resided in the Dakar region, especially in the suburbs (55.4%). After stroke, the majority of patients were first referred to the primary health care center (n=22 or 39.3 %). Only 14% of patients (n=8) firstly presented to a university hospital. Prehospital transfer was primarily made by cab (53.6%, n=30), private car (37.5%, n=21), nonmedical ambulance (5.4%, n=3) and ambulance (3.6%, n=2). More than half of the patients (67.9% or n=38) had a past medical history. Hypertension was the most common history (73.7%; n=28). Approximately two-thirds of the patient had a risk factor in their history, 31.5% (n=12) had two or more associated risk factors and 18.4% (n=7) had diabetes and hypertension and no history of stroke (Table 1). The mean NIHSS score was 13.8 ± 5.75 (ranging between 3 and 24) and the median was 15.

**Table 1:** patient distribution by personal history

Previous history	Numbers of patients	Percentages of patients (%)
High blood pressure (HBP)	16	42.1
HBP + Diabetes	7	18.4
HBP + Stroke	4	10.5
Strroke	3	7.9
HBP + stroke + diabetes	1	2.6

**Figure 1:**
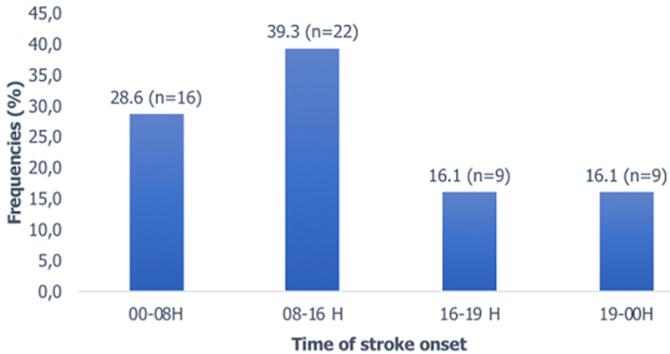
patient distribution by time of stroke onset

**Figure 2:**
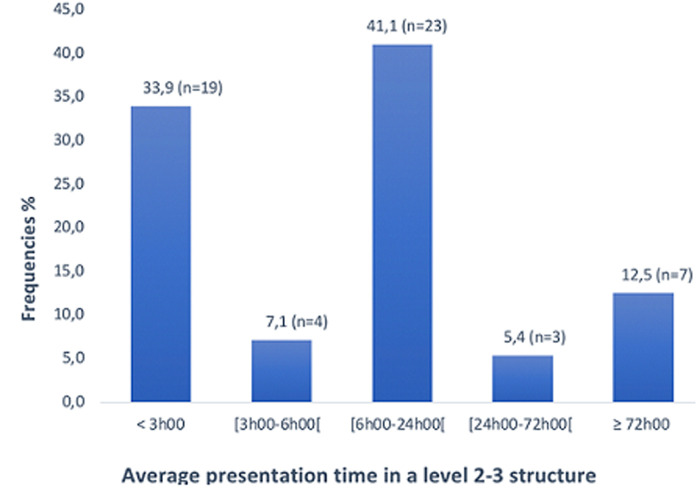
breakdown of patients according to presentation time brackets in a level 2-3 structure

The most common location of stroke onset was home (85.7%, or n=48). Other places of stroke onset were at work (8.9% or n=5), at a party (3.6% or n=2) and on the street (1.8% or n=1). Stroke occurred most often (n=22 or 39.3%) between 8 a.m and 4 p.m (Figure 1) and 64.3% (n=36) occurred on weekdays. The average seeking time interval in the pathway's to primary care setting was 6.45 hours ± 13.53 (ranging between 0.25 and 96 hours). The median was 2 hours. More than half of the patients (58.9%; n=33) presented in less than 3 hours to a primary care setting. In addition, 3 patients (5.4%) presented in more than 24 hours (Table 2). The average seeking time interval in the pathways to a level 2-3 structure was 26.59 hours ± 55.34. The median was 8.5 hours. The majority of patients presented to a level 2-3 facility within 6 hours (41% with n=23) and between 6 and 24 hours (41% with n=23) (Figure 2). The average imaging time was 28.9 hours ± 54.6 and (ranging between 1.5 and 240 hours). The median was 10.5 hours. The majority of patients underwent imaging test over the 6-24-hour period (51.8% or n=29).

**Table 2:** breakdown of patients according to presentation time brackets in a level 2-3 structure

Onset to door time range	Numbers of patients	Percentages of patients (%)
< 3h00	33	58.9
03h00-06h00	7	12.5
06h00-24h00	13	23.2
24h00-72h00	2	3.6
≥ 72h00	1	1.8

In univariate analysis, delayed presentation to a level 2-3 facility was significantly associated with female gender (84.6% p=0.006), unmarried marital status (89.5% p=0.008), and weekday stroke (77.8% p=0.013). Being followed-up for a vascular risk factor did not significantly improve the time of arrival at a level 2-3 facility after stroke. Stroke severity as determined by NIHSS was not significantly associated with delayed presentation to a level 2-3 setting. Similarly, patient treatment pathway, social network and the mean of prehospital transportation (cab, private car, ambulance) were not associated with delayed presentation to the emergency department in a level 2-3 facility. In a multivariate analysis, the determinants of delayed presentation to level 2-3 health care setting were female (OR = 5.5 (95% CI: 1.5-19.8), being married (OR = 7.2 (95% CI: 1.5-35.8), and the occurrence of ischemic stroke during weekday (OR = 4.3 (95% CI: 1.3-13.9) (Table 3).

**Table 3:** factors associated with late utilization of a level 2-3 structure

Variables associated with late recourse	Late recourse to a level 2-3 health structure				
	N	%	Total	P value	Ods aj [CI at 95]
**Gender**				0.006	
Women's	22	84.6	26		5.5 [1.^5^-^19^.^8^]
Men	15	50.0	30		Ref
**Marital status**				0.008	
Married	17	89.5	19		7.2 [1.5-35.8]
Unmarried	20	54.1	37		Ref
**Stroke day**				0.013	
Week	28	77.8			4.3 [1.^3^-^13^.^9^]
Weekend	9	45.0			Ref

## Discussion

**Onset-to-door presentation´s time:** at the end of our study, 33.9% of patients with ischemic stroke arrived at a level 2-3 hospital within three hours. In a similar inpatient study conducted in 2012, Bassong *et al*. evaluated the seeking time interval in the pathways to neurological consultation among patients with both ischemic and hemorrhagic stroke. Only 20.7% of patients with ischemic stroke arrived at the neurology consultation within three hours [12]. In their study of survival of comatose patients after both ischemic and hemorrhagic stroke in the neurology department of Fann, Sène *et al*. found that 27.8% of patients with ischemic stroke consulted within 3 hours of deficit onset [13]. Our study focused on assessing the time interval in the pathways to a level 2-3 hospital among patients with acute ischemic stroke. The study populations were therefore different from those of other studies conducted in the department. However, we noted an increase in the proportion of patients who presented early to a level 2-3 facility after ischemic stroke. This could be the result of successful public health and media campaigns on awareness of neurovascular disease, together with the explosion of social networks that reach more and more people. However, considering the idea of establishing a neurovascular unit (NVU), only 33.9% of patients would be eligible for revascularization by intravenous thrombolytic therapy (rt-PA) provided they meet the other eligibility criteria, which are both clinical and paraclinical [14,15]. In contrast, 71.4% and 41% of our patients presented to both a primary care facility and a level 2-3 medical facility within 6 hours of stroke, respectively. Lacy *et al*., reported that 61% of patients had a post-stroke emergency department visit within six hours [14]. Although 71.4% of patients presented to a health care facility relatively early, only 57.5% arrived at a level 2-3 facility in less than six hours.

This could be the consequence of the national health policy, which establishes the therapeutic itinerary of patients in such a way that they have to resort to primary health care structures in the unexpected event of the occurrence of a health problem. If we consider the time it takes to complete brain imaging test for diagnosis, only 10% of patients had their imaging completed in less than 3 hours. This further reduces the number of people who would have been eligible for the rt-PA program [14,15]. As a result, approximately 20% of patients who arrived at the emergency department within 3 hours were diagnosed with ischemic stroke beyond the time required to be eligible for thrombosis. Nearly eight out of ten patients arrived after 3 hours and 41% took between 6 hours and 24 hours to get to a level 2-3 hospital´s emergency department after their ischemic stroke. This implies that the majority of our patients would immediately reduce their chances of being eligible for thrombosis if a NVU is established [10,11,14,16]. For our patients, the median time of presentation to the emergency department was 8.5 hours, although the mean time was more than 24 hours because some patients presented after 10 days. These numbers translate the fact that prehospital delay is very high for the majority of patient with acute ischemic stroke. Jin *et al*. had a median greater than ours (12 hours) [17] and Wester *et al*. had a lower median (5.1 hours) [18]. These variations can be explained by the different structures of the cities in which the studies were conducted and also by the varying accessibility of transportation, since our study was conducted in Dakar, a smaller urban center than the one in which Jin conducted his study but our patients had a prehospital transportation system less robust than patients in Wester´s study.

**Determinants of late presentation:** in our study, we identified three non-modifiable factors associated with late referral. The first of these was patient gender: women were 5.5 times more likely than men to arrive late. In contrast, other studies [12,14,18] showed that gender was not significantly associated with delayed presentation to the emergency department. This can be explained by the cultural and social differences between our environment and theirs. Indeed, in our environment, women are very often in a vulnerable situation [19] because they are financially dependent on a third party and this third party, if absent at the time of the onset, is often expected to make the decisions of attending hospital, and he started on their way to the hospital. The second unmodifiable factor significantly associated with delayed access to the emergency room was marital status: univariate analysis showed that being single was significantly associated with delayed access, while, multivariate analysis showed that married people were 7.2 times more likely to arrive late. In Jin *et al*. study, univariate analysis, showed that being married was significantly associated with early arrival; however, multivariate analysis showed that there was no significant relationship between marital status and the time of presentation of their patients [17]. Our results can be explained by the fact that in our society, decision-making often depends on who pays for the expenses related to care. If this subject was not present at the time of stroke, which predominantly occurred at home and while at work, the decision to go to the emergency department was very often delayed, even though the time and location of stroke onset was not associated with a high prehospital delay. The final factor associated with the late presentation was the day of stroke onset: patients with a stroke occurring on weekdays were 4.3 times more likely to experience delays than those with stroke occurring during the weekends. This may be related to the fact that the family was often together during the weekends and, as the majority of strokes occurred at home, the response time was shorter and patients arrived earlier than in the cases in which the stroke occurred during the weekdays. Social network and family structure have a definite virtuous role, more for men than for women, in patients presentation to the emergency department. Early presentation to a level 2-3 emergency department during the weekends may also be due to less traffic and shorter travel times to the hospital for patients who live predominantly in the suburbs.

In our study, age was not associated with delayed presentation. In Lacy *et al*. [14] study, multivariate analysis showed that age greater than 65 years was associated with early admission. He attributed this to greater knowledge and awareness of stroke among older adults. Our results may be due to the fact that our population was much younger than those in other similar studies [14,16-18], with a mean age of 49 years and a median of 45 years and 73% of patients under 60 years of age. Patient itinerary (which facility was visited first and mode of transportation), prognosis of NIH Stroke Scale/Score(NIHSS), location of onset, time of onset, place of residence, whether or not in paid employment, and type of employment were also not associated with delayed presentation. Other authors have shown a significant association between patients' itinerary and their time of arrival at a level 2-3 facility [12,14,20]. This may be due to a higher level of public awareness of stroke. The absence of a referral structure and the limited human and material resources dedicated to prehospital management of neurovascular emergencies in our environment may also explain why our patients' therapeutic itinerary was not significantly associated with late recourse. We did not find any relationship between late referral and place of residence. In Bassong *et al*. study, the place of residence was significantly associated with patients' late recourse: living outside the Dakar region was associated with late recourse [12]. Our results could be explained by the fact that our study was conducted during a period of interregional travel restrictions related to the COVID-19 pandemic. Patients' history (hypertension, stroke, diabetes) was not associated with delayed presentation to a level 2-3 hospital. This is likely due to the fact that education about the risks of ischemic stroke does not specifically target patients with risk factors but target the general population. Joachim Fladt *et al*. [20] observed that a patients' history of hypertension and diabetes were associated with an early arrival, but that having a history of stroke did not reduce time to presentation. Overall, our results suggest that reducing delays in emergency department visits after ischemic stroke is necessary to improve patients´ vital and functional prognosis. In fact our population was much younger than that described in other countries [12,15] and the majority of our patients were eligible for thrombolysis according to the NIHSS. The forthcoming establishment of neurovascular units and references neurovascular units (RNVU) in Senegal would only be truly effective if action is taken against the modifiable factors that affect patients' eligibility for thrombolytic treatment, which are, on the one hand, the time of presentation and factors associated with it and, on the other hand, the time required to perform brain imaging test. In our study, we did not identify factors associated with delayed CT, but these should be analyzed in order to improve management of ischemic stroke. Our study provides decisive information to those involved in neurovascular pathology to improve patient management.

**Limitations:** in spite of their significance, these results cannot be extrapolated to the population, as this study has some limitations, including the relatively small sample size. This can be explained on the one hand by the six-month study period and on the other hand by the occurrence of the COVID-19 pandemic, which was associated with a reduction in usual hospital activities. Patients had become reluctant to come to our hospital, which was the referral center for the management of COVID-19 cases. A larger sample could provide additional information on the other variables studied in determining factors associated with delay in patient presentation.

## Conclusion

Stroke is a real public health problem associated with a high burden on patients and their families. The younger the individual, the greater the burden on the family. The advent of neurovascular units and revascularization in the early phase of ischemic stroke has helped improve the prognosis. However, in sub-Saharan Africa, few countries have neurovascular units or referrals neurovascular units. Our study shows that the majority of patients targeted for thrombolysis present late to the emergency departments of hospitals as well as to house neurovascular units. It also shows that the number of patients with early-onset stroke is significantly higher than in previous similar studies. Non-modifiable factors such as female gender, married marital status, and stroke onset during a weekday were significantly associated with delayed presentation in a multivariate analysis which emphasized the vulnerability of women, in particular married women, in our countries.

### What is known about this topic

- *In African countries stroke is more likely to affect young-adults patients;*
- *The burden of stroke tremendously reduce by revascularization therapies;*
- *The arrival bellow six hours is one of the criterias for being eligible to revascularization therapies; increasing and reinforcing prehospital management's capabilities of stroke patients reduces the delay from onset to needle*.

### What this study adds

- *Gives a picture of prehospital habit of patients with stroke in our country;*
- *Highlights the vulnerability of women mostly married women in our countries;*
- *Strongly advocates for creating neurovascular units across sub-Saharan African countries*.
